# Exendin-4 Improves Diabetic Kidney Disease in C57BL/6 Mice Independent of Brown Adipose Tissue Activation

**DOI:** 10.1155/2020/9084567

**Published:** 2020-02-03

**Authors:** Shu Fang, Yingying Cai, Fuping Lyu, Hongbin Zhang, Chunyan Wu, Yanmei Zeng, Cunxia Fan, Shaozhou Zou, Yudan Zhang, Ping Li, Ling Wang, YaomingXue, Meiping Guan

**Affiliations:** ^1^Department of Endocrinology & Metabolism, Nanfang Hospital, Southern Medical University, Guangzhou, Guangdong, China 510515; ^2^Women and Children's Hospital, Xiamen, Fujian, China 361003; ^3^Department of Endocrinology and Diabetes, The First Affiliated Hospital, Xiamen University, Xiamen, China 361001; ^4^Department of Biomedical Sciences, University of Copenhagen, Copenhagen, Denmark 2200; ^5^Department of Endocrinology and Metabolism, Hainan General Hospital, Haikou, Hainan, China 570311; ^6^Department of Endocrinology, Dongguan TungWah Hospital, Guangdong, China 523900

## Abstract

**Background:**

The role of exendin-4 in brown adipose tissue (BAT) activation was not very clear. This study is to verify the role of BAT involved in renal benefits of exendin-4 in diabetes mellitus (DM).

**Methods:**

In vivo, C57BL/6 mice were randomly divided into nondiabetic (control) and diabetic groups (DM). The diabetic mice were randomized into a control group (DM-Con), BAT-excision group (DM+Exc), exendin-4-treated group (DM+E4), and BAT-excision plus exendin-4-treated group (DM+Exc+E4). The weight, blood glucose and lipids, 24 h urine albumin and 8-OH-dG, and renal fibrosis were analyzed. In vitro, we investigated the role of exendin-4 in the differentiation process of 3T3-L1 and brown preadipocytes and its effect on the rat mesangial cells induced by oleate.

**Results:**

The expressions of UCP-1, PGC-1*α*, ATGL, and CD36 in BAT of DM mice were all downregulated, which could be upregulated by exendin-4 treatment with significant effects on ATGL and CD36. BAT-excision exacerbated high blood glucose (BG) with no significant effect on the serum lipid level. Exendin-4 significantly lowered the level of serum triglycerides (TG) and low-density lipoprotein- (LDL-) c, 24 h urine albumin, and 8-OH-dG; improved renal fibrosis and lipid accumulation; and activated renal AMP-activated protein kinase (AMPK) in diabetic mice regardless of BAT excision. In vitro, there was no significant effect of exendin-4 on brown or white adipogenesis. However, exendin-4 could improve lipid accumulation and myofibroblast-like phenotype transition of mesangial cells induced by oleate via activating the AMPK pathway.

**Conclusions:**

Exendin-4 could decrease the renal lipid deposit and improve diabetic nephropathy via activating the renal AMPK pathway independent of BAT activation.

## 1. Introduction

Diabetic kidney disease (DKD), one of the serious complications of diabetes, has becoming the leading cause of end-stage renal disease (ESRD) all over the world [1, 2]. Not only can hyperglycemia lead to DKD, obesity-induced lipid deposition can also lead to the renal cells exposed to high concentration of free fatty acids (FFA) [3], which is associated with microvascular and matrix remodeling in the kidney disorders such as DKD.

Glucagon-like peptide-1 (GLP-1), an incretin hormone, improves glucose metabolism through promoting secretion of insulin, inhibiting secretion of glucagon, and gastric emptying [4, 5]. Recent studies indicated that exendin-4, a GLP-1R agonist (GLP-1RA), could improve DKD through multiple mechanisms [6–8]. Brown adipose tissue (BAT) can also improve obesity and its related diseases. Activating BAT improved nephropathy in diabetic mice induced by high-fat diet (HFD)/streptozotocin (STZ) [9, 10]. It has been reported that bone morphogenetic protein 7 (BMP7) and AMP-activated kinase (AMPK) could regulate the signal pathway of the development of BAT, which also play a key role in the kidney disorders [11, 12]. BAT has been verified as an endocrine organ to regulate the metabolism of distant organs [13]. However, it is unknown yet whether the beneficial effect of exendin-4 on diabetic nephropathy is dependent on BAT activation.

Considering exendin-4 in the activation of brown adipose tissue (BAT), a promising approach against obesity and diabetes, is distinctly reported in several literatures. We mainly explored the renal beneficial effects and its mechanism of exendin-4 in DM and whether this effect is achieved by directly acting on BAT.

## 2. Materials and Methods

### 2.1. Cell Cultures

Rat mesangial cell lines (HBZY-1, MCs) were cultured in complete DMEM culture medium (HyClone, MA, USA), 10% fetal bovine serum, penicillin, and streptomycin. The cells were incubated at 37°C in a humidified 95% air, 5% CO_2_ atmosphere-designated incubator. Original oleic acid or palmitic acid (both from Sigma-Aldrich, Santa Clara, CA, USA) were added to mimic the FFA-albumin complexes in obesity. MCs were cultured in DMEM medium alone for 12 hours for synchronization and then were exposed to FFA-BSA complexes (200 *μ*mol/L), with or without the additional application of exendin-4 (0.1, 1, 10, 20, or 100 nM), compound C (10 *μ*M), or AICAR (1 mM). The MTT (Cell Signaling Technology, Danvers, MA, USA) method was used to examine the cell proliferation.

Mouse 3T3-L1 preadipocytes were obtained from the Chinese Academy of Sciences, and the brown preadipocytes were established as previously described [14]. First, we isolated the interscapular BAT of C57BL/6, then we use collagenase to digest the BAT. Differentiation of preadipocytes was carried out according to the protocol which has been described in detail in the previous study [15, 16]. Cells were incubated with or without exendin-4 (10 nmol/L) from day 0 to day 8. Furthermore, the differentiated cells were cultured with serum-free DMEM after differentiation, and 12 hours before treatment, the cells were then treated with exendin-4 (10 nmol/L) or vehicle.

### 2.2. Animal Experiments

Male C57BL/6J mice (six weeks old) were purchased from the Guangdong Medical Laboratory Animal Center. All mice were nurtured under controlled conditions (temperature 22°C, 12-hour light-dark cycle) in the specific pathogen-free (SPF) barrier facility at the Southern Medical University.

### 2.3. Mouse Models of T2DM

The male C57BL/6J mice were randomized to the chow diet (CD, *n* = 8) or high-fat diet (HFD, 60% of total calorie from fat, *n* = 39) groups. After 4 weeks, the HFD group was made diabetic by STZ (120 *μ*g/g body weight, pH 4.5; Sigma S0130) diluted in 10 mmol/L sodium citrate buffer; the control mice were infused with the same amount of citrate buffer. The mice were judged to be diabetic when the blood glucose exceeded 250 mg/dL. Then, the diabetic mice were divided into 4 groups (*n* = 8-10 per group): diabetic mice (DM-Con), BAT-excision diabetic mice (DM+Exc), exendin-4-treated diabetic mice (DM+E4), and exendin-4-treated diabetic mice with BAT excision (DM+Exc+E4). The BAT was removed from the intrascapular region of the diabetic mice in the DM+Exc group and the DM+Exc+E4 group. Exendin-4 (sigma E7144) was administered to DM+E4 mice and DM+Exc+E4 mice by intraperitoneal injection for 8 weeks, and the DM-Con mice received the injection of saline. Mice were euthanized after 12 weeks, and the kidneys were obtained for further analyses.

### 2.4. Biochemical Measurements

Blood glucose levels were measured using an Accu-Chek glucose monitor (Bayer Corp.) every week. After mice were euthanized, collected blood and the serum was stored at -80°C. The concentrations of triglycerides (TG), HDL-cholesterol (HDL-C), and LDL-cholesterol (LDL-C) were measured by commercial kits (Nanjing Jiancheng Bioengineering Institute, Nanjing, China).

Twenty-four-hour urine collections for measurements were obtained from mice placed in metabolic cages. Urinary microalbumin concentrations and the levels of 8-hydroxy-2′-deoxyguanosine (8-OH-dG) were measured using enzyme-linked immunosorbent assay (ELISA) kits from Bethyl Laboratories, Inc., Montgomery, TX, USA, and CUSABIO, USA, respectively.

### 2.5. Quantitative Real-Time PCR and Western Blot Analysis

Total RNA was extracted with TRIzol (Takara, Dalian, China), and the RNA quality was detected by the NanoDrop ND-1000 spectrophotometer (Thermo Fisher Scientific Inc., MA, USA). The cDNA was synthesized by M-MLV Kit (Invitrogen, Carlsbad, CA, USA). The SYBR Green qPCR kit (Takara, Dalian, China) was used to perform real-time quantitative PCR (RT-qPCR) in the Roche LightCycler 480 II system (Roche, Basle, Switzerland). The expression of the transcript is normalized by the expression level of *β*-actin.

RIPA lysis buffer (Beyotime, Shanghai, China) was used to extract the total protein of the sample, and the protein concentration was measured by a BCA assay (Takara, Dalian, China). The protein was separated by 10% SDS-PAGE (Bio-Rad, Hercules, CA, USA) and transferred to the PVDF membrane (Merck Millipore, MA, USA). Blocking with 5% skim milk in TBST for 1-2 hours, the PVDF membrane was incubated with primary antibodies overnight at 4°C. The primary antibodies were as follows: phospho-adenosine 5′-monophosphate- (AMP-) activated protein kinase (AMPK) *α* (Thr172) and AMPK*α*, phospho-acetyl-CoA carboxylase (ACC) (Ser79) and ACC, transforming growth factor- (TGF-) *β*1 (all from Cell Signaling Technology, Danvers, MA, USA), collagen I (Col-1) and fibronectin (FN) (both from Abcam, Cambridge, MA, USA), and *α*-smooth muscle actin (SMA) and *β*-actin (both from Boster Bio Co., Wuhan, China).

### 2.6. Histological Analysis

Oil Red O (Sigma-Aldrich) staining was used to determine TG accumulation as previously described [17]. The total TG of MCs was extracted and measured by a TG Assay Kit (Applygen Technologies Inc., Beijing, China). After being fixed for 48 h by 4% paraformaldehyde, the renal tissue was dehydrated and then embedded in paraffin, cut into 4 *μ*m thick slices, and stained with periodic acid-Schiff (PAS) and Masson's trichrome. All sections were detected by Olympus B ×40 upright light microscope (Olympus, Tokyo, Japan).

### 2.7. Statistical Analysis

All data were represented as the mean ± SD. The differences between two independent groups were examined by two-tailed Student's *t*-test, and the differences among three independent groups were examined by one-way ANOVA. Statistical analysis was performed by using SPSS 20. There was a significant difference when *P* < 0.05.

## 3. Results

### 3.1. Exendin-4 Improved Glucose and Lipid Metabolism in Diabetic Mice

**Figure 1:**
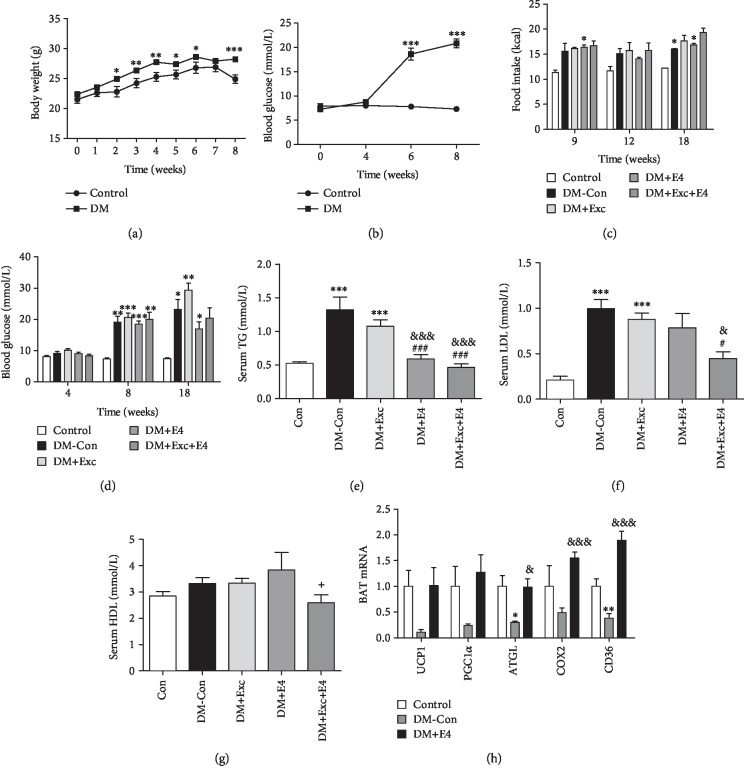
Exendin-4 treatment improved metabolism in diabetic mice induced by high-fat diet (HFD) and STZ. The body weight (a), random blood glucose (b, d), and food intake (c) were recorded during the period of building the model of diabetic mice. The serum levels of TG (e), LDL (f), and HDL (g) were measured by ELISA. BAT-specific genes (h) were analyzed. *n* = 6‐8 for each group. ^∗^*P* < 0.05, ^∗∗^*P* < 0.01, and ^∗∗∗^*P* < 0.001 vs. control group; ^#^*P* < 0.05 and ^###^*P* < 0.001 vs. DM+Exc group; ^&^*P* < 0.05 and ^&&&^*P* < 0.001 vs. DM-Con group; ^+^*P* < 0.05 vs. DM+E4 group.

After 8 weeks, the body weight and blood glucose of diabetic mice increased significantly compared with those of the control group (Figures 1(a) and 1(b)). However, there was no significant difference between the two groups with regard to food intake (Figure 1(c)). The blood glucose level of the DM+Exc group was significantly higher than that of the other groups. Exendin-4 treatment could restore this effect to some extent with no significance (Figure 1(d)). To explore the effect of exendin-4 on lipid metabolism, we measured the contents of TG, LDL, and HDL. DM-Con mice and DM+Exc mice showed great higher levels of TG and LDL, which could be reversed by exendin-4 treatment (Figures 1(d)–1(g)). But there was no significant effect on the HDL level.

The expressions of peroxisome proliferator-activated receptor gamma coactivator 1-alpha (PGC-1*α*), uncoupled protein-1 (UCP-1), cluster of differentiation 36 (CD36), and adipose triglyceride lipase (ATGL) in BAT of DM mice were all downregulated. Exendin-4 treatment could upregulate ATGL and CD36 significantly and nonsignificantly increased the expression of UCP-1 and PGC-1*α* (Figure 1(h)).

### 3.2. Exendin-4 Ameliorated the Progress of Diabetic Nephropathy and Activated Renal AMPK Pathway

**Figure 2:**
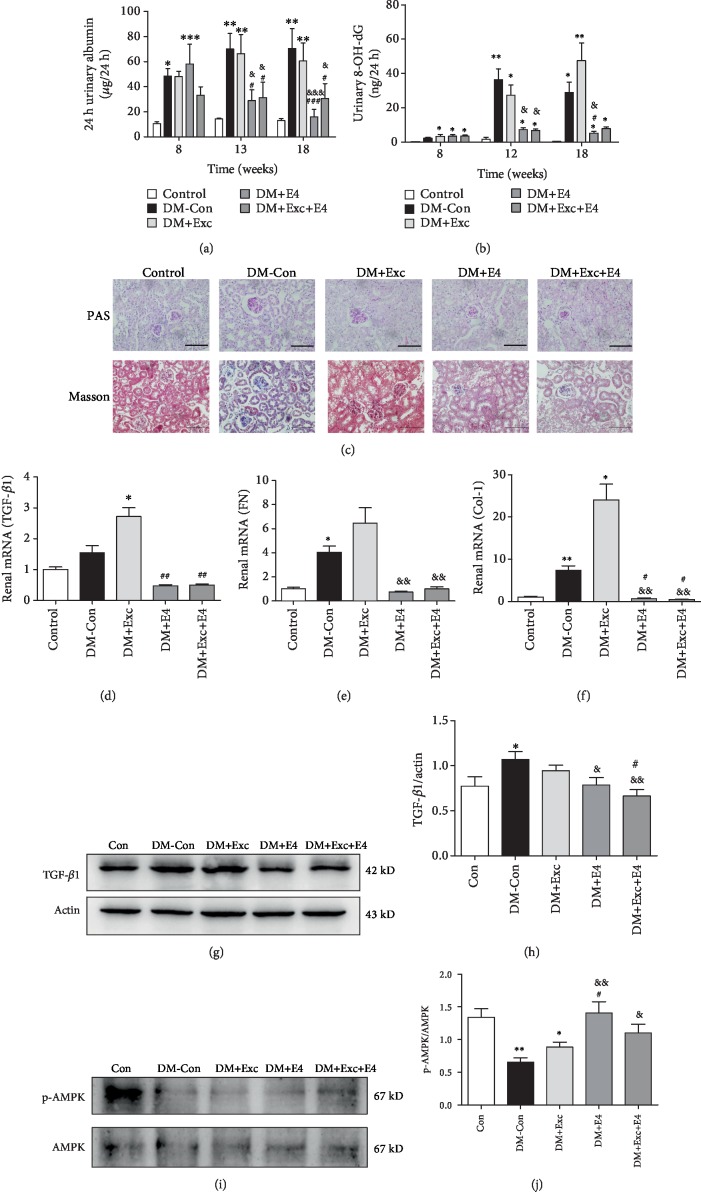
Exendin-4 ameliorated the progress of diabetic nephropathy and activated the renal AMPK pathway. The 24-hour urinary albumin (a) and 24-hour urinary 8-OH-dG (b) were measured by ELISA. (c) Periodic acid-Schiff (PAS) and Masson staining of kidney. Magnification ×400. (d–f) The renal mRNA expression of TGF-*β*1, Col-1, and FN, respectively (*n* = 6 per group). Western blot analysis of renal TGF-*β*1 (g, h) and AMPK (i, j) protein and the quantitative analysis results (*n* = 5 per group). *n* = 6~8 per group. ^∗^*P* < 0.05, ^∗∗^*P* < 0.01, and ^∗∗∗^*P* < 0.001 vs. control group; ^#^*P* < 0.05, ^##^*P* < 0.01, and ^###^*P* < 0.001 vs. DM+Exc group; ^&^*P* < 0.05, ^&&^*P* < 0.01, and ^&&&^*P* < 0.001 vs. DM-Con group.

Exendin-4 decreased urinary albumin excretion and the urinary 8-OH-dG level (Figures 2(a)–2(b)). Diabetic mice also showed obvious glomerular mesangial expansion; the PAS and Masson staining indicated that the increase in DM-Con mice and DM+Exc mice was suppressed by exendin-4 (Figure 2(c)). We also detected the mRNA abundance of renal TGF-*β*1, Col-1, and FN to explore the renal fibrosis in mice, a major pathological change in DKD. The mRNA of TGF-*β*1, Col-1, and FN (Figures 2(d)–2(f)) and the protein of TGF-*β*1 (Figures 2(g) and 2(h)) were significantly increased in DM-Con mice and DM+Exc mice. And exendin-4 completely reduced these increases. These results demonstrated that BAT excision could aggravate the damage of renal function, and exendin-4 treatment could improve this damage.

AMPK regulates a series of physiological events, including mitochondrial function and cellular growth [18, 19]. Our previous study found that exendin-4 could alleviate rat mesangial cell proliferation induced by high glucose through decrease of the phosphorylation of extracellular regulated protein kinases (ERK) and the expression of rapamycin (mTOR) via AMPK activation [20]. In this study, we found that renal AMPK phosphorylation was significantly decreased in diabetic mice, which could be improved by exendin-4 treatment (Figures 2(i) and 2(j)).

### 3.3. Exendin-4 Has No Significant Effect on Adipocytes In Vitro

**Figure 3:**
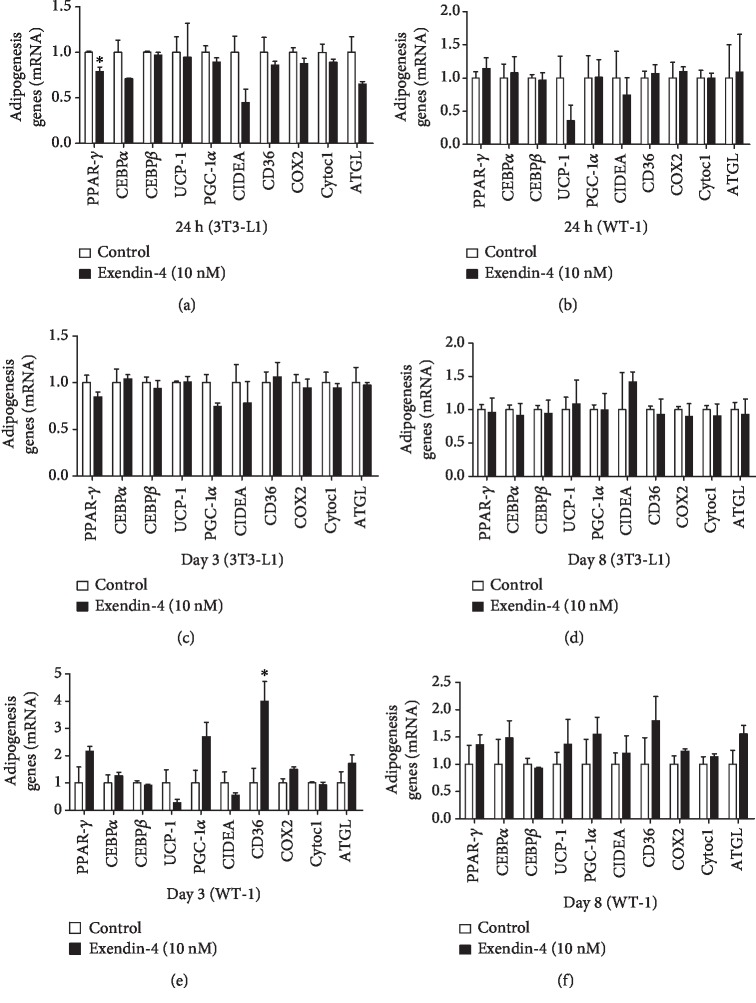
Effects of exendin-4 on adipogenesis-related genes during differentiation of 3T3-L1 and brown preadipocytes (WT-1). The mRNA expression of PPAR-*γ*, CEBP*α*, CEBP*β*, UCP-1, PGC-1*α*, CIDEA, CD36, COX2, Cytoc1, and ATGL in mature 3T3-L1 cells (a) and brown preadipocytes (b) with exendin-4 (10 nM) stimulation for 24 hours and days 3 and 8 of differentiation in 3T3-L1 cells (c, d) and brown preadipocytes (e, f), stimulated by exendin-4 (10 nM) (*n* = 3 per group). ^∗^*P* < 0.05 vs. control.

We further analyzed the effect of exendin-4 on white and brown adipogenesis in vitro. The expressions of UCP-1, PGC-1*α*, Cell Death-Inducing DFFA-Like Effector A (CIDEA), peroxisome proliferator-activated receptor- (PPAR-) *γ*, CCAAT Enhancer Binding Protein alpha (CEBP*α*), CEBP*β*, CD36, cyclooxygenase (COX) 2, cytochrome (Cytoc) 1, and ATGL were determined during the differentiation process of 3T3-L1 and brown preadipocytes. The results showed that only PPAR-*γ* was significantly downregulated by exendin-4 in mature 3T3-L1 cells (Figure 3(a)) and that there was no effect in brown mature adipocytes (Figure 3(b)). During the differentiation, only CD36 was upregulated by exendin-4 at day 3 in WT-1 cells (Figures 3(c) and 3(d)), and there was no significant effect on the differentiation of 3T3-L1 (Figures 3(e) and 3(f)). Thus, exendin-4 did not exert obvious effect on brown adipogenesis and activity in vitro.

### 3.4. Exendin-4 Improved the Myofibroblast-Like Phenotype Transition in MCs Induced by Oleate

**Figure 4:**
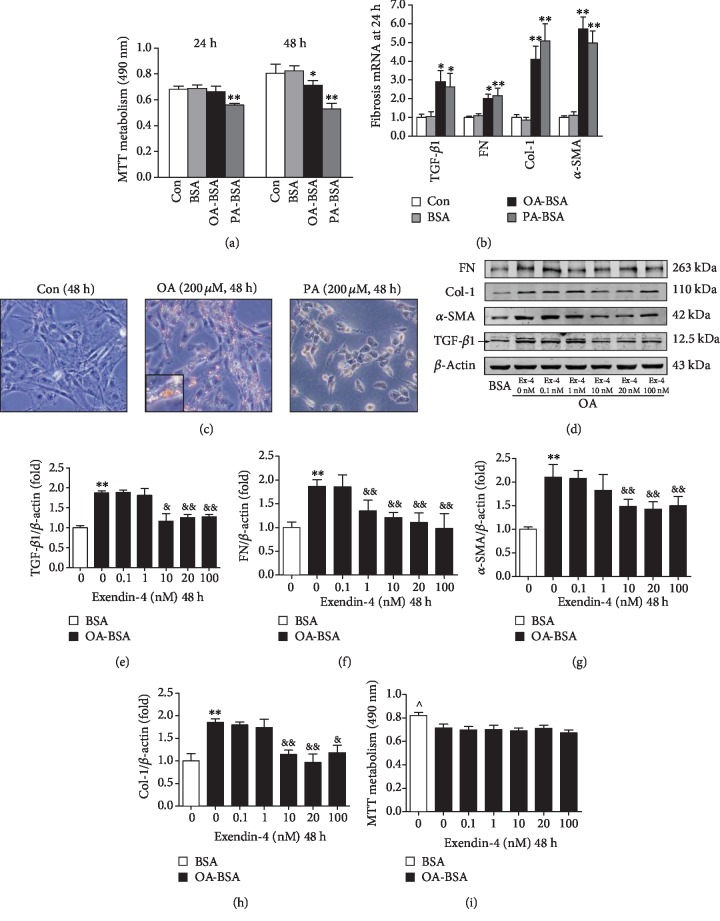
Effect of FFA-BSA complexes on myofibroblast-like phenotype transition and lipid accumulation in cultured mesangial cells. Quiescent mesangial cells were treated with media (control, Con), 1% fatty acid-free BSA (BSA) alone, 200 *μ*M oleate- or palmitate-1% fatty acid-free BSA (OA-BSA and PA-BSA, respectively), or exendin-4 of 0 (vehicle control), 0.1, 1, 10, 20, and 100 nM for 48 hours. (a) Proliferation of MCs by MTT assay. (b) Expressions of TGF-*β*1, FN, *α*-SMA, and collagen I by quantitative RT-PCR. (c) Lipid accumulation in MCs determined by Oil Red O assay. Inset presents ×10 magnification to illustrate size and location of lipid droplets in cytoplasm. (d) Western bolt analysis of TGF-*β*1, FN, *α*-SMA, and collagen I. Quantitative analyses of the results are also shown: TGF-*β*1 (e), FN (f), *α*-SMA (g), and Col-1 (h). Values are the mean ± SD of 3 independent experiments. ^∗^*P* < 0.05 and ^∗∗^*P* < 0.01 vs. BSA group, ^&^*P* < 0.05 and ^&&^*P* < 0.01 vs. OA-BSA with 0 nM exendin-4 group. (i) Dose response of MC viability in response to increasing concentration of exendin-4. ^^^*P* < 0.05 vs. other groups.

The cell activity measured by MTT metabolism was decreased by 13.8 ± 0.8% in the oleate group at 48 hours compared to the BSA group (Figure 4(a)). Oil Red O staining revealed that exposure to oleate greatly increased the size and quantity of neutral lipid droplets in the cytosol, while the body of cells stimulated with palmitate shrank and became rounded with pyknotic nuclei compared to that of the control (Figure 4(c)). Both mRNA (Figure 4(b)) and protein (Figure 4(d)–4(h)) expressions of TGF-*β*1, FN, *α*-SMA, and Col-1 were significantly increased in the oleate group compared with the BSA control, while this increase is suppressed by treatment with 10 nM and 20 nM of exendin-4. There were no significant changes observed in the cell viability between groups (Figure 4(i)).

### 3.5. AMPK Pathway Involved in the Effects of Exendin-4 on Myofibroblast-Like Phenotype Transition and Lipid Accumulation in MCs

**Figure 5:**
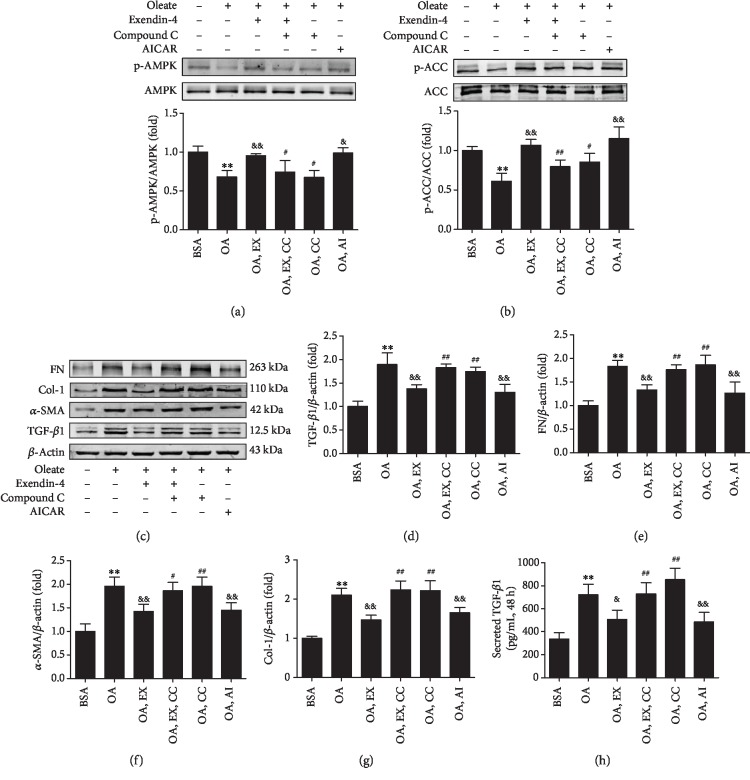
Exendin-4 regulates the expressions of TGF-*β*1, FN, *α*-SMA, and Col-1 in MCs treated by oleate via AMPK activation. EX: exendin-4; CC: compound C; AI: AICAR. MCs were cultured in DMEM and 1% BSA or 200 *μ*M oleate-BSA for 48 hours. Exendin-4 (20 nM), compound C (10 *μ*M), or AICAR (1 mM) were used to assess the effect of AMPK on myofibroblast marker protein expression. Supernatants were assayed by ELISA after treatment with a different reagent. Western bolt of p-AMPK (a), p-ACC (b), TGF-*β*1, FN, *α*-SMA, and Col-1 (c) and quantitative analyses of the results are also shown (d–g). (h) Secreted TGF-*β*1 in the cell culture supernatants. Values are the mean ± SD of 3 independent experiments. ^∗∗^*P* < 0.01 vs. BSA group; ^&&^*P* < 0.01 vs. OA group; ^#^*P* < 0.05 and ^##^*P* < 0.01 vs. OA+EX group.

The phosphorylation of AMPK and ACC was inhibited in MCs cultured with oleate, while 20 nM of exendin-4 significantly restored their phosphorylation (Figures 5(a) and 5(b)). The effect of exendin-4 was reversed by compound C. On the contrary, AICAR had a similar effect as exendin-4. AICAR and exendin-4 significantly inhibited oleate-induced protein expression of fibrogenic markers (Figures 5(c)–5(g)), which can be reversed by compound C. Moreover, exendin-4, as well as AICAR, significantly decreased the TGF-*β*1 secretion induced by oleate in cell culture supernatants, which was also reversed by compound C (Figure 5(h)).

**Figure 6:**
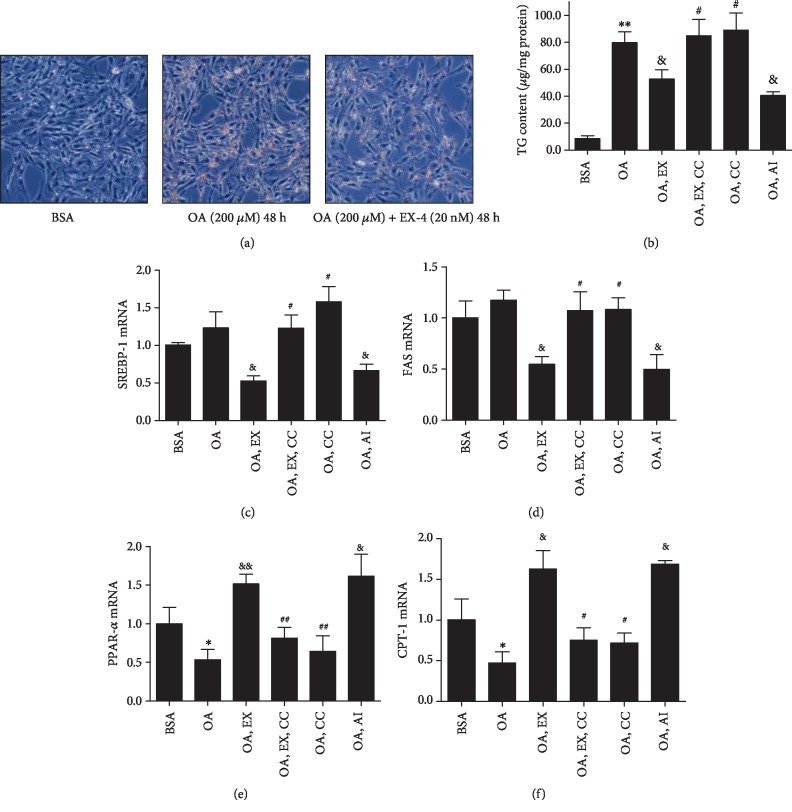
Effects of exendin-4 on lipid accumulation and mRNA expression of SREBP-1, FAS, PPAR-*α*, and CPT-1 mediated by AMPK activation. Exendin-4 (20 nM), compound C (10 *μ*M), or AICAR (1 mM) were used to assess the effect of AMPK in MCs cultured with 1% BSA or 200 *μ*M oleate-BSA. Deposition of neutral lipid was characterized by Oil Red O (a), and triglyceride (TG) contents of the cell were measured by glyceridase assay (b). The mRNA expressions of SREBP-1, FAS, PPAR-*α*, and CPT-1 were measured by quantitative RT-PCR (c–f). ^∗^*P* < 0.05 vs. BSA group; ^&^*P* < 0.05 and ^&&^*P* < 0.01 vs. OA group; ^#^*P* < 0.05 and ^##^*P* < 0.01 vs. OA+EX group, *n* = 3.

Oleate greatly increased the lipid accumulation in MCs which can be decreased by exendin-4 (Figure 6(a)). A glyceridase assay was used to accurately reflect TG changes in MCs. The result showed that exendin-4 inhibited oleate-induced TG accumulation significantly (Figure 6(b)), which can be attenuated by compound C. The expressions of SREBP-1 and FAS were inhibited by exendin-4 and AICAR (Figures 6(c) and 6(d)), which were reversed by compound C. Oleate downregulated the expressions of PPAR-*α* and CPT-1, which were reversed by exendin-4 (Figures 6(e) and 6(f)).

## 4. Discussion

Exendin-4, a GLP-1RA, has already been used for several years to treat patients with type 2 diabetes, especially obese patients. In trials designed for cardiovascular outcomes, GLP-1RAs, such as liraglutide and semaglutide, were associated with a relevant reduction in the incidence and progression of nephropathy [21, 22]. In STZ-induced diabetic rats, continuous i.p. infusion of exendin-4 decreased renal expression of the proinflammatory factor, reduced albuminuria, and blocked glomerular and tubulointerstitial fibrosis [23]. Exendin-4 treatment significantly ameliorated glomerular hyperfiltration, extracellular matrix formation, inflammation, and apoptosis in db/db mice [7]. These studies indicated that exendin-4 could not only improve the glucose and lipid metabolism, but also ameliorate the pathological process of diabetic nephropathy. In our study, exendin-4 improved glucose and lipid metabolism and renal fibrosis in diabetic mice. In vitro, treatment with exendin-4 significantly reduced the expression of matrix proteins in a dose-dependent manner in rat mesangial cells induced by oleate. GLP-1 has a direct role against renal oxidative stress by inhibition of NAD(P)H oxidases through cAMP-protein kinase A (PKA) pathway activation [24, 25]. The beneficial effect of exendin-4 on human MCs cultured in high glucose is largely dependent on the activation of adenylate cyclase [26]. In our study, exendin-4 inhibited TGF-*β*1 and myofibroblast-like phenotype transition of MCs induced by oleate via AMPK pathway activation.

AMPK, a metabolic sensor of ATP in the cells, has been implicated as a target for correcting metabolism, especially in the kidney [12, 27]. Activated AMPK regulates cellular responses to low energy states and coordinates the activity of enzymes of lipid metabolism [28]. Moreover, it has been widely reported that AMPK attenuated hepatic lipogenesis through phosphorylation of downstream ACC and downregulation of the related enzymes associated with lipid metabolism [29]. Similarly, our study showed that exendin-4 increased phosphorylation of AMPK and ACC, which correlated with decreased TG contents in the cultured MCs. Oleate inhibited the expression of PPAR-*α* and CPT-1, while exendin-4 reversed all of these changes, indicating that the renal benefits of exendin-4 can be mediated by promoting lipolysis and inhibiting lipogenesis via AMPK pathway activation.

Activation of brown adipose tissue (BAT) is a promising method to combat obesity and metabolic diseases, such as T2DM and dyslipidemia, which not only depends on thermogenesis but also on secretory function [13, 30, 31]. Previous studies reveal that BAT transplantation could improve obesity and hyperglycemia in mice [9, 10]. Exendin-4 activated central GLP-1R to increase plasma TG and glucose clearance in HFD-induced mice via BAT activation and WAT browning mediated by the hypothalamic AMPK pathway [32]. Considering that the intracerebroventricular infusion of exendin-4 was different from our intraperitoneal injection and the distributions of GLP-1R, these could lead to distinct consequences. In this study, exendin-4 treatment upregulated the expression of UCP-1, PGC-1*α*, ATGL, and CD36 in BAT of DM mice; the expression of these genes was downregulated in DM mice. However, exendin-4 could improve metabolism and renal lipid deposit regardless of whether BAT is removed. Lately, a randomized, double-blind, placebo-controlled trial shows that there was no effect of exenatide (exendin-4) administration on measures of energy expenditure (EE) or substrate oxidation in nondiabetic subjects with obesity [33]. However, some studies reported that GLP-1 promoted the expression of PPAR-*γ* and CEBP*α* in 3T3-L1 adipocytes [34]. In our study, exendin-4 did not show directly the effect on adipocytes in vitro but it could improve the activity of BAT in DM mice. It seems more likely that cerebral GLP-1R is involved in BAT activation.

In conclusion, exendin-4 treatment could decrease renal lipid deposits and improve diabetic nephropathy via activating the renal AMPK pathway regardless of the BAT status.

## Data Availability

The data used to support the findings of this study are available from the corresponding authors upon request.
